# Personal and Work-Oriented Characteristics Distinguishing Older Nurses’ Partial or Complete Actual Retirement Behavior over Three Years

**DOI:** 10.3390/ijerph20146348

**Published:** 2023-07-12

**Authors:** John Rodwell

**Affiliations:** Department of Management & Marketing, Swinburne University of Technology, Hawthorn, VIC 3122, Australia; jrodwell@swin.edu.au

**Keywords:** retirement behavior, age, partly retiring, bridge employment, older nurses

## Abstract

To retain nurses and prevent worsening the nursing shortage, a key opportunity is to better understand the drivers of complete and partial retirement of older nurses. This study investigates the characteristics that distinguish older nurses’ partial and complete actual retirement behavior, from those continuing to work, over a three-year period. A quantitative longitudinal design comprising 217 female Australian nurses aged 50 years or over, from two samples working at Time 1 (2012 and 2016), responding three years later (Time 2). Multinomial regression found two different patterns of drivers for each of completely retiring and partly retiring respectively. Age was the only variable distinguishing both partly and completely retired nurses from nurses who were not retired. The further variables distinguishing completely retired nurses were not being prosperous, having impaired work ability, being partnered, not stressed at work and working part-time. The only variable beyond age distinguishing partly retired nurses was having a casual contract. Offering flexible work options in terms of working hours and contracts that suit the nurse’s lifestyle and supporting nurses with health impairments to continue working are options that may lessen the number of nurses completely retiring and instead either remaining at work or partly retiring.

## 1. Introduction

The shortage of nurses acknowledged by the first report published by the World Health Organization (WHO) on the State of the World’s Nursing [1], projected a shortfall of 10.6 million nurses by 2030. The International Council of Nurses (ICN) has since suggested that the shortage may be more like 13 million nurses [2]. Notably, these numbers may be conservative estimates because they excluded countries, primarily high-income countries, with staffing levels above a certain benchmark. If the high-income countries wanted to maintain or increase their nursing levels, then the shortage may be considerably higher, a situation exacerbated by the international mobility of nurses from low- and middle-income countries to high-income countries.

In the context of global nursing workforce shortages, there is a growing imperative to keep the skills and expertise of older nurses in the workforce. For example, in Australia the proportion of the nurse workforce older than 45 years of age increased from 29% in 1996 to 47.4% in 2005 [3], plateauing at around 51.7% in 2015 [4]. In the context of the ageing of the population and the ageing of the nursing workforce, Australia will face a shortage of nurses over the coming years, particularly due to retirement [5]. Most studies of nurses’ withdrawal from work have investigated turnover (e.g., [6]), but studies are needed on the drivers of nurse retirement specifically [7]. A key opportunity for addressing these shortages is through the increased supply of nurses from delayed retirements [8].

The investigation of the retirement of nurses also needs contemporary studies due to the changing nature of nursing and the changing nature of retirement. The last few decades have seen retirement transition processes change from emphases on complete retirement to increasingly flexible processes [9], including bridge employment, signifying the ‘bridge’ from pre-retirement employment to full retirement [10].

Managers wanting to avoid shortages could try to keep nursing staff for longer, delaying their complete retirement and/or facilitate the bridging to retirement of nurses. Consequently, this study will investigate predictors of actual retirement behavior, whether partial or complete, for working nurses 50 years of age or older over a three-year time span.

### 1.1. The Life Course Theoretical Framework

The life course perspective is a theoretical framework that is capable of describing the longitudinal adjustment process of retirement [11]. Life course theory is a sociocultural perspective on age often used to explain retirement decisions through the consideration of normative age, prescribed transitions, age expectations, and age patterns over the life course [12]. In particular, the life course perspective adds the consideration of social time as expressed in the age patterning of social roles and careers. That is, an individual’s life course is structured by social influences and the life choices made in constrained situations [13].

The situational constraints are not necessarily the main drivers. Rather, human agency is embodied by the life choices made under constrained situations, where the resulting behavior varies depending on the timing in that person’s life. That is, the life course perspective is a multidisciplinary framework for investigating the dynamics of diverse, interdependent paths to retirement where individuals make choices and plans from among available options [14,15]. For example, bridge employment allows older workers to choose their level of workforce participation [16], allowing a gradual transition and adjustment from work to full retirement [17].

When considering the issues that impact retirement decision making, many of the issues are grouped in a decision framework such as that of Feldman [17], where information is assessed regarding relevant work and non-work variables often categorized in terms of “push” and “pull” factors. Perhaps more importantly though, most prior retirement research has been cross-sectional, yet in the life course perspective retirement is a process that takes place over time and therefore should be studied longitudinally [14]. Yet, there are a range of variables that have yet to be tested longitudinally with a focus on actual retirement behaviors, particularly for specific contexts such as nursing.

### 1.2. Predictors of Retirement Behaviors for Nurses

The lack of research investigating drivers of nurses’ retirement behavior, particularly whether partial or complete, entails that the literature reviewed below will often have to extrapolate from life course theory and studies of intent to retire in order to derive possible drivers. Studies of predictors of nurses’ retirement, or similar issues, have been heterogeneous and can be grouped in terms of age and personal factors, or organizational factors [18]. This study uses a similar broad grouping of issues.

Life course theory suggests that the options individuals have the agency to choose from are driven by transition-relevant individual attributes, which may include demographic characteristics, health, and financial status [11]. Similarly, the personal characteristics investigated here include two of the stronger predictors of retirement consideration: finances, then health and work ability. The remaining key personal issues are age, gender and family issues such as marital status and having children, as well as education. The organizational characteristics considered here include global assessments of satisfaction with work, stress from work, type of contract and whether full-time or part-time.

Finances have been argued to be a key reason for people retiring, but the more specific measure of salary has been found to not be related to the early retirement of nurses [7]. Broad analyses across many studies suggest that finances, usually cast in terms of income, are not a dominant motivator for nurses to extend their working life [18]. Arguments that nurses may remain at work when they feel they need the income, may also be a function of the rank of the nurse [8]. Yet, large scale analyses found that rank and type of institution also did not predict nurses’ behavior (retirement combined with turnover), even with income not in the analyses [19]. Conversely, once nurses reach a level where they feel financially secure, they may be more likely to intend to retire [20].

The pattern of results, where income may or may not be a predictor of retirement thoughts and may be a reflection of rank or context, suggests that something about finances may be a driver of retirement, but not income specifically. The relative importance of financial comfort reflects assessments of the current life situation considering possible dependency on an age pension and financial responsibilities in the context of life stage and cohort-historical factors [12]. An additional factor is the retirement income system in Australia, which is a three-pillar system consisting of voluntary savings, compulsory savings (held inside a retirement account), and a publicly funded age pension [21,22]. Australia adopted a compulsory retirement saving system in 1992 where employers paid a gradually increasing percentage of the employee’s income into a retirement account [22]. Despite the relatively long duration of saving, many retiring today did not make compulsory contributions during early career stages and have insufficient savings to maintain their desired living standards [23].

Another factor which may impact retirement decisions is the age at which retirement savings can be accessed. Savings accumulated in a retirement account can be partially accessed (e.g., 4–10% yearly) once a person reaches preservation age, which for most people would currently be 60 years. At that point they can access their funds as a lump sum (ceasing employment) and/or via withdrawals akin to an annuity that could supplement a reduction in paid hours worked [24].

An income can also be derived from the publicly funded age pension after reaching 67 years of age. The payment is means tested against income (from savings or employment) and assets, while also adjusted for couples and singles [25]. Compared to other OECD countries, the age pension in Australia is very low, replacing only 41% of pre-retirement earnings when a full age pension is received [26]. As retirement savings are inadequate for many retirees, at least a partial age pension is paid to around 62% of people aged over 65 [27].

The retirement income system also has structural barriers to retirement saving for certain cohorts which may impact retirement behavior. For example, females are particularly disadvantaged under the existing system, as compulsory saving contributions cease during extended periods out of the labor force, such as when raising children [28]. Females typically retire with less savings as a result, although those working in the public sector have historically received more retirement savings contributions than those in the private sector [29]. Moreover, as income is correlated with education [30,31], those with higher levels of educational attainment also tend to have more retirement savings which may lead to earlier retirement.

Perhaps after considering these issues, such as eligibility for pensions or accessing superannuation, the key is that nurses make a judgement as to whether they will be financially secure (in a similar manner to propositions by [7] and others). That is, nurses are making an assessment as to whether they are prosperous enough that they can afford to retire. Therefore, rather than judging specific incomes or financial incentives, this study will assess nurses’ financial security in terms of perceived sufficiency of prosperity as a potential driver of retirement behavior.

After financial concerns, nurses often indicate that their next main consideration in thinking about retirement is their health [18,20]. Health is often not significant in cross-sectional studies predicting retirement intentions or preferences if those studies do not include measures of disability (e.g., [7,8]). A large-scale study of predictors of nurses’ actual behavior (retirement combined with turnover) in Italy over 12 months did find that poor health and disability predicted behavior for older nurses although those specific health elements were part of a multi-dimensional measure of work ability [19].

The ability to perform work due to health or work ability decreases in terms of functional capacity as workers age, impacting work participation until retirement [32]. Further, health conditions are associated with increasing rates of workplace injury in nursing may limit work ability [33]. Therefore, with health conditions and/or disability making it difficult to continue to work, the effects of health on retirement behavior may occur over time and need to be studied longitudinally.

### 1.3. Personal Drivers of Retirement Behavior

The next group of personal drivers reflect social characteristics of the consideration of retirement, particularly age, gender, and the family considerations of marital status and children. Age and its meanings provide an essential dimension in life-course study by linking age to time, context, and process [13]. The social meanings of age structure the life course perspective through age expectations, informal sanctions, and normative social timetables that can influence transitions such as retirement [13,14]. That is, individuals may feel that they have little choice over whether to retire, or feel social constraints, especially if they want to retire ‘late’ or beyond a normative age [34]. Similarly, as nurses age they feel these social pressures and think about the timing of their retirement [18], but the specific consideration of age’s impact on retirement behavior is complicated because age is often built in to many studies’ definitions of retirement. Therefore, studies need to consider age itself, when age is not part of the retirement variable, such as through consideration of partial and complete retirement behaviors.

Gender has been found to be associated with nurses’ intentions for early retirement [7]. Yet analyses of the drivers of actual behavior for older nurses found no effect of gender [19]. Not least among the possible reasons for this non-result of gender is that nursing staff are predominantly female, a contextual issue that often necessitates a focus on female nurses. Further, familial caring duties or their social situation may be a driver of women being more likely to retire early, perhaps because they are married, with two incomes, and thus are financially secure enough to retire, or in order to care for dependent children. To separate out these possible effects, each of these drivers (e.g., prosperity, marital status, children) are specifically considered.

In the family context, marital status and raising children have had inconsistent effects on retirement. Nurses with a spouse have been found to retire earlier than single nurses [7]. Yet the results for divorcees and widows is less consistent [8], although that inconsistency may be due to smaller sample sizes when using those more specific categories. Similarly, little is known about the effects of children being present on retirement. In the related area of retirement’s associations with health, children in the home when nearing or at retirement may impact health by promoting health-enhancing regimens, or may lead to less effort on self-care [35]. The pattern of results across these and other studies suggests that it may be worthwhile investigating whether the nurse is partnered and whether children are present and dependent.

There is little consensus on whether educational attainment is associated with retirement behavior. Changes in educational policies and requirements for nurse registration in many countries may mean that earlier studies, or meta-analyses of those earlier studies, may not be applicable now, where nurses have a broader and higher level of formal education, suggesting more research is needed.

### 1.4. Work-Oriented Drivers of Retirement Behavior

The next section moves on to the work-oriented drivers of retirement behavior. These work characteristics range from relatively specific components of work, such as low levels of autonomy, lack of flexible hours, and heavy workloads through to broader issues such as the pace of technological change (e.g., see [36,37]). Instead of detailing a wide range of work characteristics, many of which may not apply to some nurses’ workplaces, an alternative and common approach is to consider the nurses’ evaluation of the combined impact of those characteristics as reflected by global measures such as work-related well-being (stress and anxiety) and job satisfaction.

Older nurses’ decisions to retire early are influenced by stress and exhaustion from work [36]. The stress experienced by nurses is due to the impact of demands such as workload that are not ameliorated by available resources [38]. That is, work-oriented poor mental health, such as in terms of anxiety, may contribute to nurses retiring [39].

Similarly, job satisfaction reflects global assessments across many working conditions such as flexible work, choice of working hours [8], ineffective supervisory relationships and poor opportunities for professional development [40]. In nursing, job satisfaction has been repeatedly found to predict intent to quit or remain [18,40,41], but few studies have investigated the utility of job satisfaction in predicting retirement behavior.

One large-scale study of behavior (retirement combined with turnover) found that job satisfaction did not predict actual behavior [19]. The non-significant result for job satisfaction may have been due to shared variance between variables such as job satisfaction and emotional exhaustion with elements of their work ability index, which included psychological resources and assessments of mental demands of work [19]. Thus, in order to investigate the specific potential relationship between job satisfaction and retirement behavior there is a need to use specific measures of potential confounds such as work ability.

Studies of nurse withdrawal from the workforce have focused on intentions rather than actual behavior [18,42]. With the study of retirement behavior to include consideration of whether nurses partly or completely retire, this study also includes structural variables that may be related to bridging to retirement such as whether work was full- or part-time or whether their contract was relatively stable (permanent or fixed) or variable (i.e., casual). Overall, this study will investigate key personal and organizational characteristics that distinguish partial and complete retirement behaviors for working nurses over three years.

## 2. Materials and Methods

The participants were from the Household, Income, and Labor Dynamics in Australia (HILDA) survey [43], a broad, publicly available, longitudinal survey. The initial sample of HILDA was intended to be representative of Australian households, excluding the military and institutions such as prisons, and uses ongoing processes to try to maintain the representativeness of the panel over time (see [43] for details). The subset of the HILDA data analyzed in this study were selected for specific time periods, to be nursing qualified, working in health services of an appropriate age.

The sample used in this study was comprised respondents who had participated in the HILDA survey at two pairs of survey waves, three years apart. The first wave pair were those who had responded at both 2012 (Time 1) and 2015 (Time 2). The second wave pair were those respondents who had responded at both 2016 (Time 1) and 2019 (Time 2).

These time periods were chosen so as to minimize the effects of the Global Financial Crisis (GFC) on the financial situation of the respondents. Large scale social forces can change the pathways to retirement, particularly through unplanned changes with economic impacts [13]. Choosing samples some time after the GFC allows one of the nurses’ main sources of retirement funds, known as superannuation in Australia (a retirement annuity scheme somewhat akin to 401 (k) in the USA) and other sources of prosperity to have bounced back to more typical levels. Similarly, the time periods were chosen to finish before the arrival of COVID19. The net result is that the sample reflects relatively normal conditions and thereby provides either a baseline for comparison for later studies on the effects of such large-scale events on retirement behavior or is an example of normal conditions that health services may return to.

The Time 1 waves of each wave pair contained a module that asked whether their highest qualification was in the field of nursing and is only repeated every fourth year (e.g., 2012 and 2016). Similarly, the retirement module, containing detailed retirement questions, is asked on a different cycle and only every fourth year, forming the Time 2 of each wave pair (i.e., 2015 and 2019). The data are the 2019 version released in 2021.

The HILDA survey protocol only asked respondents aged 45 and over the detailed retirement questions (the only section that includes the delineation of part-retiring). Checks of the earliest age of any form of retirement found that there were no retirees in the 45 to 49 age group, with a few nurses retiring in their early 50s, suggesting a natural cut-off around age 50 where nurses began to enact retirement behavior. Any non-retired cases from the earlier wave pair who appeared in the later wave pair were removed from the early wave pair to avoid duplication and to try to ensure any retirees were compared as closely as possible to their peers of the time, at the time they were enacting the retirement decision. Further information on HILDA is available in [43] and the HILDA Survey has University of Melbourne Ethics approval number 1647030.

Australia has an aging population that is driving demand for health services, while simultaneously constraining workforce supply [44]. To work as a nurse or midwife in Australia requires having completed an approved course and be registered with the Nursing and Midwifery Board of Australia [45]. In Australia the number of nurses per 1000 population is similar to the USA, just above the OECD average and the salaries of Australian nurses are somewhat above the OECD average [46]. Although the mix between different categories of nurses varies across OECD countries, the vast majority of nurses are categorized as ‘professional nurses’ and a minority are considered to be at the lower, ‘associate professional’ nurse level in Australia, the United States of America (USA), Germany, and the United Kingdom [47]. Australia does not have mandatory retirement for nurses, nor most other occupations.

At Time 1 of each wave-pair, the participants selected were those indicating that nursing was the main field of study for their highest completed qualification. The occupations of the respondents, as indicated by their 2-digit Australian and New Zealand Standard Classification of Occupations (specifically—ANZSCO 2006), confirmed that the vast majority of the nursing-qualified respondents were working in an occupation as either Health Professionals, Health and Welfare Support Workers, or Carers and Aides, with the remainder being in relevant managerial or educational categories. That is, most of the sample were working in direct health care provision, although seven were in corporate/managerial roles, three were in other nursing-related professional roles (e.g., social and welfare) and one in a role with an education emphasis. The numbers of the sample in managerial roles were not sufficient to analyze or control for as a variable. Doing the analyses without the managers made no difference to the pattern of significant results. Further, due to the very low proportion (approx. 7%) of male respondents, only females were retained in the sample.

### 2.1. Measures

The measures associated with work or that had some volatility were measured at Time 1. Those measures that were slow changing, such as marital status, resident children, and highest level of education were assessed at Time 2. Marital status and education in particular did change for a few respondents between Time 1 and Time 2. Studies for higher degrees take multiple years and would not show up on the measure below until complete, and, similarly, getting divorced can take some time. Consequently, the Time 2 measures for these slow-changing variables were seen as more accurate indications of their Time 1 predictive status, where people would have known that they were studying for a higher degree or getting divorced at Time 1, but that that imminent change had not yet been completed and would not have shown up on the variables below until Time 2. The target variable of retirement status is as at Time 2, three years after the first set of questions.

Age—participants indicated their age last birthday at Time 2 (30th June 2015 or 2019).

Prosperity—financial well-being at Time 1. Participants were asked about their perceived prosperity. The Prosperity item used in HILDA had been tested as part of the International Social Science Survey, Australia (IsssA) 2000 [43]. The question was: “Given your current needs and financial responsibilities, would you say that you and your family are”, with the options of (1) Prosperous, (2) Very comfortable, (3) Reasonably comfortable, (4) Just getting along, (5) Poor, or (6) Very poor. Responses were grouped 1–3 (More prosperous), relative to 4–6 (Not prosperous).

General Health at Time 1. General health was assessed by the global question from the SF-36 [48]: “In general, would you say your health is” with response options of Excellent, Very good, Good, Fair, and Poor. The responses were coded such that excellent and very good were combined, good was separate, and fair and poor were combined.

Work Ability at Time 1. Respondents indicated whether they had a long-term health condition, disability, or impairment from a list they were shown. The list asked about disabilities/health conditions that have lasted, or are likely to last, 6 months or more, restrict everyday activity, and cannot be corrected by medication or medical aids. The response option, for the whole list overall, was a single Yes or No. Those respondents answering yes were also asked whether the condition limits the type or the amount of work they could do, with responses of Yes, No, or Unable to do any work. Those answering yes to this follow-up question were asked to indicate how much their condition(s) limit(s) the amount of work they can do on a scale from zero to 10. An answer of 0 means “not at all” and an answer of 10 means “unable to do any work”. Those respondents answering No to the initial question, or No to the limiting question, or 0 to the degree of impact question were classed as 0 No work ability impact, whereas those going through the other filters that ended up scoring 1–10 on the degree of impact item were coded as 1 work ability impacted.

The work ability variable was based on a sequence of questions with, first, the health condition or disability status question text that comes from the General Customer Survey of the (Commonwealth of Australia’s) Department of Family and Community Services. The list of activities used to define disability, however, comes from the Australian Bureau of Statistics’ Survey of Training and Education [43]. Second, the item regarding the impact of disability or condition on work is similar to a question asked in many surveys, including the British Household Panel Study (BHPS) and the (US) Panel Study of Income Dynamics (PSID). Third, the item about how much the condition limits work is similar to questions asked in many surveys (e.g., the BHPS and the PSID), although an 11-point rating scale is used in HILDA [43].

Work ability has been measured in several ways by previous studies, such as a 10-point scale [49], not being on sick leave [50], sick days taken in past month [51], and the Work Ability Index (WAI) [52]. A key feature of the WAI is the combination of both subjective and objective responses, relying on a combination of self-rated work ability questions, and questions related to sick days and disease types. The measure employed in this study is whether a person’s functional capacity to work is reduced by a medical condition. The questions used to construct this measure have similarities to items 3 and 4 of the WAI [52], and the 10-point scale used by [49]. Data availability prevents the inclusion of remaining items included in the WAI.

Marital Status at Time 2. The marital status of the respondent at Time 2 was obtained by asking: ‘Looking at [the options below], which of these best describes your current marital status?’ The response options were: 1 Married (in a registered marriage), 2 Separated, but not divorced, 3 Divorced, 4 Widowed, 5 Never married but living with someone in a relationship, and 6 Never married and not living with someone in a relationship. The codes were grouped such that 1 and 5 were classed as Partnered and the other options as Not Partnered/Single.

Resident Children at Time 2. The respondents were asked how many children they had ever had or adopted and how many of these children live in the household at least 50% of the time, or live in a non-private dwelling (building school, university hall of residence, institution) but spend the remainder of their time with you, or the number of resident step/foster/grandchildren with no resident natural/adopted parent. If resident children were indicated for any of the three feeder questions, the variable was coded 1 Yes, 2 No.

Education—Highest level of education achieved as at Time 2. Participants were asked their highest year of school they’d completed and since leaving school what qualifications had they completed, excluding hobby or recreation courses. Across the responses the highest level of education was coded and then grouped: Year 11 of school or less, Year 12, or Certificate 3 or 4, Diploma or Advanced Diploma (Less than or equal to/LTE Diploma). The other group included those with a Baccalaureate/Bachelor’s degree, Graduate Diploma or Postgraduate (PG) degree such as a Master’s degree or doctorate (Graduate and PG degrees).

Work-driven Job Stress or Anxiety at Time 1. Two items delineating job stress or anxiety (per [53]) were used that asked respondents about their current (main) job. The stress item asked the extent to which the respondent agreed that “[M]y job is more stressful than I had ever imagined”. The anxiety item asked respondents to indicate the extent to which they agree that “I fear that the amount of stress in my job will make me physically ill”. Each of these two items was rated from Strongly Disagree (1) to Strongly Agree (7). For the job stress question, the responses were grouped as 1–3 (not stressed), relative to 4–7 (stressed). The job anxiety responses were grouped 1–2 (not anxious), relative to 3–7 (anxious).

Job Satisfaction at Time 1. Respondents chose a number between 0 totally dissatisfied and 10 totally satisfied to indicate “All things considered, how satisfied are you with your job?” For the analyses responses were grouped 0-8 (Less satisfied), relative to 9–10 (Satisfied).

Contract Type at Time 1. Participants were asked which of certain categories best describes your current contract of employment? The options were ‘Employed on a fixed-term contract’, ‘Employed on a casual basis’, or ‘Employed on a permanent or ongoing basis’. The fixed-term and permanent responses were combined in contrast to the casual responses.

Working Hours at Time 1. To ascertain hours worked, participants were asked: “Including any paid or unpaid overtime, how many hours per week do you usually work in all your jobs? This includes any work performed at the workplace and at home. Don’t include time on-call”. However, if the respondent’s working hours were indicated as being variable, they were asked to give the average number of hours worked per week over the last four weeks. The resulting number of hours worked was coded as being either less than 35 h per week (<35 h), or greater than or equal to (GTE) 35 h per week.

Work-Retirement Status at Time 2. Respondents were asked: Do you consider yourself to be completely retired from the paid workforce, partly retired, or not retired at all? Responses were coded as Completely retired (3), Partly retired (2), or Not Retired at All (1).

### 2.2. Data Analysis

The final sample had 237 female, nursing-qualified respondents aged 50 and over, working in health-related occupations, matched from Time 1 to Time 2 across both wave pairs, although 20 cases had one or more variables with missing data. Missing values analyses found that the missing values were missing completely at random (MCAR) with Little’s *p* = 0.806.

Multinomial regression was performed using SPSS 26 on to retirement status at Time 2 based on 217 longitudinal responses following [54]. The descriptive statistics for the variables analyzed by each of the Retirement Status categories are in Table 1. At Time 2 there were 160 nurses who remained not retired, 26 had partly retired, and 31 had completely retired. Most of the respondents had permanent or fixed contracts. A majority of the nurses worked part-time hours, were not so satisfied with their work, were partnered, and did not have resident children, respectively. A substantial majority were in good or excellent health and did not have a disability or health condition that impacted their work ability.

The full multinomial regression model had −2LL = 228.810, a significant improvement over the intercept only base model of 327.105 (χ^2^ (26) = 98.295, *p* < 0.001), indicating that the predictors, as a set, distinguished between the three retirement categories at Time 2. The Nagelkerke R-square = 0.467 and the Cox and Snell R-square = 0.364. Table 2 summarizes the logit parameter estimates (with their Standard Errors) and the Odds Ratios with their 95% confidence intervals. The comparison end state for the multinomial regression was where the respondents were not retired at Time 2.

## 3. Results

Age was the only variable that significantly distinguished both partly retired and completely retired respondents from those in the not retired group. The variables that distinguished those who completely retired, other than age, were low levels of stress, work ability, being partnered, and not being as prosperous. Notably, work ability distinguished completely retiring, but not partly retiring, from the not retired. That is, if the nurses had no health conditions or work ability issues, they were more likely to not retire than completely retire. Other than age, the only variable that distinguished the respondents who partly retired was contract type, where casuals were more likely to partly retire.

The results were checked by pooled multinomial regression across 40 multiple imputation datasets. The results found had the same significant relationships, with the exception that the significant relationship of partnered status on completely retired in Table 2 dropped to *p* < 0.10. Consequently, the partnered finding may be considered unstable.

Further checks were performed in terms of permutations of the Age variable, such as including the age pension eligibility age (65 in Australia at that time) using a dichotomous variable categorizing respondents as aged less than 65, or 65 or older at Time 2. The check variable was not significant and did not change the pattern of results in Table 2.

## 4. Discussion

The strength and consistency of age in distinguishing both partly and completely retired respondents from the not retired group appears to support the idea that the normative pressures discussed by [34] may make some older nurses feel that they have to retire. These findings support [18], but extend their results to show that the normative effect also appears to apply to part retirement. Notably though, the age effect was linear, rather than a function of age pension eligibility and the age effect remained above and beyond any effect of other variables such as work ability.

Finding that low levels of stress characterized those nurses who completely retired extends previous studies that found that stress (e.g., [37]) impacted decisions to retire early. The stress effect could be due to either low demands and/or having more than enough resources to ameliorate those demands (per [38]), suggesting a boredom effect, or a lack of challenge, leading to nurses choosing to completely retire.

Specific measures of health impairments impacting work ability predicts the specific behavior of completely retiring, extending [19]. Impaired work ability predicting completely retiring may be a reflection of decreasing functional capacity for the older nurses (per [32]) and/or reflect the accumulated impacts of injuries on work ability [33]. Conversely, extending the conclusions of [20] from retirement intentions to retirement behavior, if the older nurses did not have any health conditions or work-ability issues they were more likely to remain at work. Notably, work ability characterized completely retiring, but not partly retiring.

The relationship of prosperity with being completely retired was that the less prosperous were tending to be more likely to be completely retired. A possible explanation for this result is that Australia has relatively universal health care and a means-tested age pension where all retirees with less than a certain level of wealth and income would be eligible for the pension. That is, less prosperous retirees may be more likely to be eligible for the full pension, whereas prosperous retirees may not be eligible for the pension at all and may want to keep working in order to maintain a certain lifestyle. Studies of non-nursing employees found that income does not predict early retirement intentions in the Netherlands, a country that also has a strong welfare context [55]. Thus, the results for finance and prosperity may be partly due to welfare context effects.

A potentially related finding was that being partnered distinguished those who completely retired, where most of the female nurses who were partnered would have older, male partners. In trying to resolve these two findings, taking low prosperity and being partnered together suggests that the prosperity variable is more about the individual nurses’ prosperity, whereas being partnered may include consideration of their partner’s financial resources. That is, individually less prosperous nurses would be more likely to not bother continuing to do work that does not advance their prosperity and instead choose to completely retire. Whereas it is more worthwhile for those who are more individually prosperous to continue working to maintain a desired lifestyle.

More broadly, the findings suggest some delineation of the life course perspective as may apply to female, older nurses. That is, in considering the context components of life course theory’s links between age, context, and process [13], particular layers and aspects of context may be important. At the national level, the extent and strength of welfare support are important considerations for retirement and the nature of the person’s lifestyle is important, particularly in terms of their desire for flexibility. These more specific and nuanced aspects of context may inform future research, especially with relatively feminized workforces.

The only variable other than age that distinguished the female nurses who partly retired was contract type, where casuals were more likely to partly retire. More specifically, those female nurses on a casual contract at Time 1 were more likely to partly retire at Time 2, essentially remaining at work with a relatively flexible work pattern into retirement. That is, having already established flexible work arrangements, and working at organizations that allow such arrangements, may reflect how the nurses may have a lifestyle that prefers that flexibility, and the nurse has become used to flexible work that fits in to that lifestyle. In terms of the transition-relevant attributes that are the drivers of agency in life course theory [11], having only age and being a casual worker distinguish part retirement from continuing to work, suggests that partial retirement may not be as clear cut a ‘transition’ as complete retirement, which had several distinguishing variables.

The social meanings of age were key for both forms of retirement behavior. In the life course perspective retirement can be seen as being influenced by social meanings of age through age expectations, sanctions, and normative social timetables [13,14]. The net result of these influences is that many nurses retire before the age pension eligibility age. Although sanctions may be thought of in terms of pension-related issues such as eligibility age, the direct drivers appear to be more in terms of expectations and judgements of future financial viability and prosperity relative to that welfare context. In a feminized workforce such judgements include normative forces that may have a more direct impact on a partner, where female nurses are likely to be younger than their usually male partners who would often have more wealth built up due to having fewer interruptions to their careers. In the long term, as social norms change, encouraging workers to remain in the workforce and as other social policies and forces have their affects over time (e.g., through wider use and utility of child-caring supports), those social forces will facilitate more nurses remaining in the workforce.

A notable set of results in this study are the variables that were not significant, especially job satisfaction. Job satisfaction is a more transitory, affective attitude and may not have distinguished either form of retirement because this study was longitudinal and/or because of the focus on actual retirement behavior. Retirement is a process that takes place over time and therefore should be studied longitudinally [14]. Consequently, future research may wish to confirm, just as [19] and this study have, whether relatively short-term oriented assessments, such as job satisfaction, are not distinguishing of retirement behavior over time.

Perhaps the main limitation to the results above is that Australia has a relatively strong welfare support system, with relatively universal access to health services and a pension system. The size of the pension has not been updated in some time and is covered by age eligibility, as well as being limited to those below specific income and wealth ceilings, effectively limiting the pension to the less well-off, the less prosperous.

A further limitation could be that this study was based on females only. However, the majority of the nursing workforce in Australia is female, so the results above would be applicable to at least the female majority of the nursing workforce.

Another possible influence on results often considered in longitudinal studies is the ‘healthy worker effect’ [39] where less healthy nurses left the workforce early, before the survey period. In this case nurses may have disproportionally left the workforce before age 50, leaving healthier nurses behind in the workforce. However, the work ability variable was still able to be found to be a significant distinguishing characteristic of those nurses who completely retired, suggesting that there was still ample opportunity for relationships between health-related variables and retirement behavior to be found.

## 5. Conclusions

There were several new contributions by this study. Perhaps the most novel contribution was the delineation of partly retiring from completely retiring behaviors, especially for nursing staff. The use of these finer gradations of retiring opens up a range of research topics and interventions that will help to retain nursing skills. Another key contribution of this study is in highlighting the need to focus on actual retirement behaviors, rather than intentions, as also called for by [42].

Although keeping nurses in the workforce on a full-time basis may be a common goal, a particularly important implication of this study’s results are to place some emphasis on partial retirement as a pathway that keeps nurses in the workforce. The direct issues that may enable nurses to partly retire are the wider use of contracts that may encourage bridging to retirement, enabling gradual transitions to full retirement (per [16,17]), timing the implementation of those mechanisms by age.

The consistency and nature of the relationship of age with the forms of retirement reflects the normative expectations of society in general, but not the specific prescribed transitions to retirement such as those embodied in eligibility for the age pension. Therefore, nurse management should not feel that their hands are tied by the proportion of their workforce over a certain age. In particular, the individual’s consideration of social roles, within the constraints of their situation, appeared to be key drivers of their choice of form of work or retirement, strongly supporting the life course perspective of [13]. With this study providing a baseline for future studies examining the impacts of situations such as the GFC or the COVID19 pandemic on the drivers of nurses’ retirement behavior.

Nurse managers may want to enact interventions that encourage both remaining at work and/or bridging to retirement. Managers may influence nurses to remain at work rather than completely retiring by addressing work ability issues and encourage staff to return to full-time hours. At a minimum such considerations could include (per [34]) health-promoting working environments, better inclusion of older workers in training at the workplace and adapting working conditions to workers, particularly to avoid injury [33]. Making work interesting and engaging, redesigning or augmenting work to support the work ability of staff, and acknowledging that staff are not chained to a need to keep their job, especially if they are married and/or in countries with strong welfare systems, are all issues for nurse managers to consider. Less prosperous nursing staff may be more likely to stay if their pay enhanced their prosperity. Over time there may be changes to the perceptions of prosperity. For example, the perceptions of the value of nursing work, as well as the shortages of nurses, may lead to increases in pay that gradually change the nurses’ perceptions of their prosperity and desire to remain working. Further, delineating forms of retirement behavior, rather than solely focusing on turnover or retirement intentions, opens up more opportunities for nurse managers to inform interventions and keep nurses working.

## Figures and Tables

**Table 1:** Descriptive statistics for the variables by work-retirement category at Time 2.

Categorical Variables		Not Retired (n)	Partly Retired (n)	Completely Retired (n)
Contract Type (Time 1)	-Casual	17	10	6
	-Permanent or Fixed	143	16	25
Working Hours (Time 1)	-Works GTE 35 h	71	5	6
	-Works < 35 h	89	21	25
Job more stressful than imagined (Time 1)	-Not much	77	15	22
	-More stressful	83	11	9
Job stress anxiety make me ill (Time 1)	-Not so much	85	16	15
	-Anxious	75	10	16
Job Satisfaction (Time 1)	-Less satisfied	115	14	20
	-Satisfied	45	12	11
Prosperity (Time 1)	-Not so much	105	21	26
	-More prosperous	55	5	5
Marital Status (Time 2)	-Not Partnered	72	12	8
	-Partnered	88	14	23
Has Resident Children (Time 2)	-Yes	69	4	10
	-No	91	22	21
Education (Time 2)	-LTE Diploma	78	8	20
	-Graduate and Postgraduate	82	18	11
General Health (Time 1)	-Poor/Fair	20	7	6
	-Good	64	6	15
	-Excellent	76	13	10
Health impact on work-ability (Time 1)	-No Impact	137	20	21
	-Some impact	23	6	10
**Continuous Variable**		**Mean (Standard Deviation)**		
Age (years)		57.41 (4.902)	62.42 (5.721)	62.00 (5.164)

Note: n = 217, GTE = Greater than or equal to, LTE = Less than or equal to.

**Table 2:** Odds ratios of characteristics distinguishing between work-retirement end points.

Work-Retirement [Ref: Not Retired at All]	Partly Retired			Completely Retired		
Variables [Reference Category]	B (SE)	Odds Ratio	95% CI	B (SE)	Odds Ratio	95% CI
Age	0.193 (0.055)	1.213 **	1.089–1.351	0.246 (0.059)	1.279 **	1.140–1.435
Contract Type = Casual [Ref: Permanent/Fixed]	1.519 (0.656)	4.566 *	1.262–16.518	0.606 (0.714)	1.833	0.453–7.424
Working Hours = GTE 35 h [Ref: <35 h]	−0.909 (0.629)	0.403	0.117–1.381	−1.345 (0.599)	0.260 *	0.080–0.843
More Stressful than imagined = Not Stressful [Ref: Stressful]	0.118 (0.747)	1.126	0.260–4.871	1.934 (0.684)	6.917 **	1.881–26.416
Stress Anxiety Make Me Ill = Not anxious [Ref: Anxious]	−0.295 (0.796)	0.745	0.156–3.544	−1.296 (0.655)	0.281 ^†^	0.078–1.014
Job satisfaction = Less satisfied [Ref: Satisfied]	−0.562 (0.598)	0.570	0.176–1.841	−0.350 (0.576)	0.704	0.228–2.178
Prosperity = Not so prosperous [Ref: More prosperous]	0.952 (0.654)	2.590	0.720–9.325	1.285 (0.647)	3.615 *	1.017–12.849
Partnered Status = Not partnered [Ref: Partnered]	−0.341 (0.547)	0.711	0.243–2.078	−1.330 (0.559)	0.265 *	0.089–0.791
Has Resident Children = Yes [Ref: No]	−0.463 (0.661)	0.630	0.172–2.302	0.409 (0.586)	1.505	0.477–4.751
Education = LTE Diploma [Ref: Graduate and Postgraduate]	−0.905 (0.548)	0.405 ^†^	0.138–1.185	0.202 (0.520)	1.224	0.442–3.393
General Health = Poor/Fair	1.318 (0.718)	3.735 ^†^	0.915–15.249	0.735 (0.781)	2.085	0.451–9.645
- Good [Ref: Excellent]	−0.154 (0.649)	0.857	0.240–3.059	0.469 (0.597)	1.598	0.496–5.148
Work ability = No impact [Ref: Some impact]	−0.834 (0.674)	0.434	0.116–1.628	−1.611 (0.619)	0.200 **	0.059–0.672
Intercept	−12.537 (3.431) **			−16.118 (3.548) **		

Note: ^†^ <0.10, * <0.05, ** <0.01, n = 217, SE = Standard Error, CI = Confidence Interval, GTE = Greater than or equal to, LTE = Less than or equal to, Ref. = Reference/comparison category.

## Data Availability

The HILDA data are available via application through the Commonwealth of Australia’s Department of Social Services Longitudinal Studies Dataverse (https://dataverse.ada.edu.au/dataverse/DSSLongitudinalStudies). The data used above were from the Wave 20 Release.

## References

[1] World Health Organization (2020). State of the World’s Nursing 2020: Investing in Education, Jobs and Leadership.

[2] International Council of Nurses (2021). The Global Nursing Shortage and Nurse Retention.

[3] Australian Institute of Health and Welfare (2006). Australia’s Health 2006.

[4] Australian Institute of Health and Welfare (2016). Nursing and Midwifery Workforce 2015: Supplementary Tables.

[5] Schofield D.J. (2007). Replacing the Projected Retiring Baby Boomer Nursing Cohort 2001–2026. BMC Health Serv. Res..

[6] Søbstad J.H., Pallesen S., Bjorvatn B., Costa G., Hystad S.W. (2021). Predictors of Turnover Intention among Norwegian Nurses: A Cohort Study. Health Care Manag. Rev..

[7] Boumans N.P., De Jong A.H., Vanderlinden L. (2008). Determinants of Early Retirement Intentions among Belgian Nurses. J. Adv. Nurs..

[8] Blakeley J.A., Ribeiro V.E. (2008). Early Retirement among Registered Nurses: Contributing Factors. J. Nurs. Manag..

[9] Szinovacz M.E., De Viney S. (2000). Marital Characteristics and Retirement Decisions. Res. Aging.

[10] Doeringer P.B. (1990). Bridges to Retirement: Older Workers in a Changing Labor Market.

[11] Wang M., Shultz K.S. (2010). Employee Retirement: A Review and Recommendations for Future Investigation. J. Manag..

[12] Elder G.H. (1975). Age Differentiation and the Life Course. Annu. Rev. Sociol..

[13] Elder G.H., Johnson M.K. (2018). The Life Course and Aging: Challenges, Lessons, and New Directions. Invitation to the Life Course: Toward New Understandings of Later Life.

[14] Fisher G.G., Chaffee D.S., Sonnega A. (2016). Retirement Timing: A Review and Recommendations for Future Research. Work Aging Retire..

[15] Hewko S., Reay T., Estabrooks C.A., Cummings G.G. (2018). Conceptual Models of Early and Involuntary Retirement among Canadian Registered Nurses and Allied Health Professionals. Can. J. Aging Rev. Can. Vieil..

[16] Adams G., Rau B. (2004). Job Seeking among Retirees Seeking Bridge Employment. Pers. Psychol..

[17] Feldman D.C. (1994). The Decision to Retire Early: A Review and Conceptualization. Acad. Manag. Rev..

[18] Markowski M., Cleaver K., Weldon S.M. (2020). An Integrative Review of the Factors Influencing Older Nurses’ Timing of Retirement. J. Adv. Nurs..

[19] Camerino D., Conway P.M., Estryn-Béhar M., Costa G., Hasselhorn H.-M. (2008). Age-Dependent Relationships between Work Ability, Thinking of Quitting the Job, and Actual Leaving among Italian Nurses: A Longitudinal Study. Int. J. Nurs. Stud..

[20] Duffield C., Graham E., Donoghue J., Griffiths R., Bichel-Findlay J., Dimitrelis S. (2015). Why Older Nurses Leave the Workforce and the Implications of Them Staying. J. Clin. Nurs..

[21] Martin B., Xiang N. (2015). The Australian Retirement Income System: Structure, Effects and Future. Work Aging Retire..

[22] Nielson L., Harris B. (2010). Chronology of Superannuation and Retirement Income in Australia.

[23] Chomik R., Piggott J. (2016). Australian Superannuation: The Current State of Play. Aust. Econ. Rev..

[24] Australian Taxation Office When You Can Access Your Super?. https://www.ato.gov.au/individuals/super/in-detail/withdrawing-and-using-your-super/withdrawing-your-super-and-paying-tax/?anchor=Whenyoucanaccessyoursuper#Whenyoucanaccessyoursuper.

[25] Services Australia Age Pension. https://www.servicesaustralia.gov.au/age-pension.

[26] OECD (2021). Pensions at a Glance 2021: OECD and G20 Indicators 2021.

[27] Australian Institute of Health and Welfare Age Pension. https://www.aihw.gov.au/reports/australias-welfare/income-support-payments-for-older-people.

[28] Best R., Saba N. (2021). Quantifying Australia’s Gender Superannuation Gap. Econ. Rec..

[29] Barrett J., Chapman K. (2001). The Australian Superannuation System. Private Pensions Systems—Administrative Costs and Reforms.

[30] Becker G.S. (2009). Human Capital: A Theoretical and Empirical Analysis, with Special Reference to Education.

[31] Mincer J. (1958). Investment in Human Capital and Personal Income Distribution. J. Polit. Econ..

[32] Soer R., Brouwer S., Geertzen J.H., Van der Schans C.P., Groothoff J.W., Reneman M.F. (2012). Decline of Functional Capacity in Healthy Aging Workers. Arch. Phys. Med. Rehabil..

[33] D’Arcy L.P., Sasai Y., Stearns S.C. (2012). Do Assistive Devices, Training, and Workload Affect Injury Incidence? Prevention Efforts by Nursing Homes and Back Injuries among Nursing Assistants. J. Adv. Nurs..

[34] Solem P.E., Syse A., Furunes T., Mykletun R.J., De Lange A., Schaufeli W., Ilmarinen J. (2016). To Leave or Not to Leave: Retirement Intentions and Retirement Behaviour. Ageing Soc..

[35] Moen P., Dempster-McClain D., Williams R.M. (1992). Successful Aging: A Life-Course Perspective on Women’s Multiple Roles and Health. Am. J. Sociol..

[36] Andrews J., Manthorpe J., Watson R. (2005). Employment Transitions for Older Nurses: A Qualitative Study. J. Adv. Nurs..

[37] Elovainio M., Forma P., Kivimäki M., Sinervo T., Sutinen R., Laine M. (2005). Job Demands and Job Control as Correlates of Early Retirement Thoughts in Finnish Social and Health Care Employees. Work Stress.

[38] Elliott K.-E.J., Rodwell J., Martin A.J. (2017). Aged Care Nurses’ Job Control Influence Satisfaction and Mental Health. J. Nurs. Manag..

[39] Olesen S.C., Butterworth P., Rodgers B. (2012). Is Poor Mental Health a Risk Factor for Retirement? Findings from a Longitudinal Population Survey. Soc. Psychiatry Psychiatr. Epidemiol..

[40] Coomber B., Barriball K.L. (2007). Impact of Job Satisfaction Components on Intent to Leave and Turnover for Hospital-Based Nurses: A Review of the Research Literature. Int. J. Nurs. Stud..

[41] Nei D., Snyder L.A., Litwiller B.J. (2015). Promoting Retention of Nurses. Health Care Manag. Rev..

[42] Halter M., Boiko O., Pelone F., Beighton C., Harris R., Gale J., Gourlay S., Drennan V. (2017). The Determinants and Consequences of Adult Nursing Staff Turnover: A Systematic Review of Systematic Reviews. BMC Health Serv. Res..

[43] Summerfield M., Freidin S., Hahn M., Ittak P., Li N., Macalalad N., Watson N., Wilkins R., Wooden M. (2020). HILDA User Manual—Release 19.

[44] Productivity Commission (2006). Australia’s Health Workforce.

[45] Australia Department of Health Nurses and Midwives in Australia. https://www.health.gov.au/health-topics/nurses-and-midwives/in-australia.

[46] OECD (2021). Health at a Glance 2021: Health Indicators.

[47] Buchan J., Black S. (2011). The Impact of Pay Increases on Nurses’ Labour Market: A Review of Evidence from Four OECD Countries. https://www.oecd-ilibrary.org/social-issues-migration-health/the-impact-of-pay-increases-on-nurses-labour-market_5kg6jwn16tjd-en.

[48] Ware J.E. (1993). SF-36 Health Survey. Manual and Interpretation Guide. Health Inst..

[49] Bobko N.A., Barishpolets A.T. (2002). Work Ability, Age and Its Perception, and Other Related Concerns of Ukraine Health Care Workers. Exp. Aging Res..

[50] Lindberg P., Vingård E., Josephson M., Alfredsson L. (2006). Retaining the Ability to Work—Associated Factors at Work. Eur. J. Public Health.

[51] Lindberg P., Josephson M., Alfredsson L., Vingård E. (2006). Promoting Excellent Work Ability and Preventing Poor Work Ability: The Same Determinants? Results from the Swedish HAKuL Study. Occup. Environ. Med..

[52] Ilmarinen J., Tuomi K. (1992). Work Ability of Aging Workers. Scand. J. Work Environ. Health.

[53] Butterworth P., Strazdins L., Rodgers B., Leach L. (2010). Deriving an Evidence-Based Measure of Job Quality from the HILDA Survey. Aust. Soc. Policy.

[54] Tabachnick B., Fidell L. (2007). Using Multivariate Statistics.

[55] Henkens K., Tazelaar F. (1994). Early Retirement of Civil Servants in the Netherlands 1. J. Appl. Soc. Psychol..

